# Genome-Wide Identification, Expression, and Functional Analysis of the Alkaline/Neutral Invertase Gene Family in Pepper

**DOI:** 10.3390/ijms19010224

**Published:** 2018-01-11

**Authors:** Long-Bin Shen, Yuan Yao, Huang He, Yu-Ling Qin, Zi-Ji Liu, Wei-Xia Liu, Zhi-Qiang Qi, Li-Jia Yang, Zhen-Mu Cao, Yan Yang

**Affiliations:** 1 Tropical Crops Genetic Resources Institute, Chinese Academy of Tropical Agricultural Sciences/Key Laboratory of Crop Gene Resources and Germplasm Enhancement in Southern China, Ministry of Agriculture, Danzhou 571737, China; LongbinShen@catas.cn (L.-B.S.); HuangHe@catas.cn (H.H.); yulingqin2017@catas.cn (Y.-L.Q.); liuziji1982@catas.cn (Z.-J.L.); weixialiu@catas.cn (W.-X.L.); ZhiqiangQi@catas.cn (Z.-Q.Q.); 2 Key Laboratory of Biology and Genetic Resources of Tropical Crops, Ministry of Agriculture, Institute of Tropical Bioscience and Biotechnology, Chinese Academy of Tropical Agricultural Sciences, Haikou 571101, China; yaoyuan@itbb.org.cn; 3 Institute of Tropical Agriculture and Forestry, Hainan University, Danzhou 571737, China; lijiayang822@gmail.com

**Keywords:** alkaline/neutral invertase, pepper, gene expression, enzymatic activity

## Abstract

Alkaline/neutral invertase (NINV) proteins irreversibly cleave sucrose into fructose and glucose, and play important roles in carbohydrate metabolism and plant development. To investigate the role of NINVs in the development of pepper fruits, seven *NINV* genes (*CaNINV1–7*) were identified. Phylogenetic analysis revealed that the CaNINV family could be divided into α and β groups. CaNINV1–6 had typical conserved regions and similar protein structures to the NINVs of other plants, while CaNINV7 lacked amino acid sequences at the C-terminus and N-terminus ends. An expression analysis of the *CaNINV* genes in different tissues demonstrated that *CaNINV5* is the dominant *NINV* in all the examined tissues (root, stem, leaf, bud, flower, and developmental pepper fruits stage). Notably, the expression of *CaNINV5* was found to gradually increase at the pre-breaker stages, followed by a decrease at the breaker stages, while it maintained a low level at the post-breaker stages. Furthermore, the invertase activity of CaNINV5 was identified by functional complementation of the invertase-deficient yeast strain SEY2102, and the optimum pH of CaNINV5 was found to be ~7.5. The gene expression and enzymatic activity of CaNINV5 suggest that it might be the main NINV enzyme for hydrolysis of sucrose during pepper fruit development.

## 1. Introduction

In higher plants, sucrose is the major product of photosynthesis and it provides carbohydrates for the development of reproductive organs (flowers and fruits) [1,2]. Sucrose is imported into the developing reproductive organs by phloem transport, and it is catalytically hydrolyzed by two different enzymes: invertase (EC 3.2.1.26) and sucrose synthase (EC 2.4.1.13) [3,4]. Sucrose synthase catalyzes the reversible conversion of sucrose to fructose and UDP-glucose, whereas invertases catalyze the irreversibly hydrolysis of sucrose into glucose and fructose [5,6]. Invertases can be distinguished by their optimum pH (acid or alkaline/neutral) [7]. Acid invertases are β-fructofuranosidases with an acidic pH optimum (pH 4.5–5.0), and are located in the cell wall or vacuole, whereas alkaline/neutral invertases belong to a novel family of glucosidases with alkaline/neutral pH optimum (pH 6.5–8.0), and are located in the cytosol or organelles [8]. Alkaline/neutral invertases were classified into two different isoforms: alkaline-Invs (optimum pH 6.5–7.0) or neutral-Invs (optimum pH 7.8–8.0) [9]. The molecular mechanism of different optimum pH between two isoforms is unclear. Many studies have described that acid invertases are involved in various aspects of the plant life cycle, including sucrose partitioning [10], environmental stimuli response [11,12], cell enlargement [13], and plant development [14]. However, the function of alkaline/neutral invertases was historically much less known, because of their low and unstable enzymatic activity [9]. Over the past few years, the alkaline/neutral invertase family genes have been identified from the genome of species, such as *Arabidopsis thaliana* [15], *Populus trichocarpa* [16], *Manihot esculenta* Crantz [17], *Vitis vinifera* [18], *Lotus japonicus* [19], *Hevea brasiliensis* [20], and *Oryza sativa* [15]. Plant alkaline/neutral invertases are divided into two different phylogenetic groups, named α and β clades [18]. In *O. sativa*, α clade members contain six exons (*OsNIN1*–*4*), while the members in β cladecontain four exons (*OsNIN5*–*8*). Notably, 10 amino acid residues within the conserved motifs were difference consistently between α and β clades of OsNINs [15]. The α clade alkaline/neutral invertases are localized in organelles (mitochondria, chloroplasts, nucleus), whereas the β clade alkaline/neutral invertases are localized in the cytosol [8]. The diversity of subcellular localization suggests that alkaline/neutral invertases have a variety of physiological functions. For instance, they have been reported to be involved in cellulose biosynthesis [21], biological and abiotic stress [22,23], shoot and root growth [24,25], germination, and flower and fruit development [8].

Pepper (*Capsicum annuum* L.) is an important vegetable and spice. In addition to its spicy flavor, the fruit is rich in natural colors and antioxidant compounds (such as vitamin C and carotenoids) [26]. Pepper is an important cash crop and it is widely used as a food flavoring substance in China. The pepper fruit is a typical sink organ. Sucrose is transported from the leaves to the pepper fruits through the phloem. Enzyme activity assay results have shown that alkaline/neutral invertases are important in determining the fate of imported sucrose during pepper fruits development [27]. The fruit set of pepper is related to the level of sugar supply [28,29]. Therefore, alkaline/neutral invertases are likely to play an important role in the formation and development of pepper fruits. However, the alkaline/neutral invertase genes of pepper have not been identified. In this study, all of the alkaline/neutral invertases were identified, based on the available sequences of the pepper genome. The evolutionary relationships, exon–intron structures, motif distributions, subcellular localizations, and three-dimensional (3D) structures of CaNINV proteins were investigated. The temporal and spatial expression patterns of *CaNINV1–7* were analyzed by RNA-seq data. Finally, the alkaline/neutral invertase activity of CaNINV5 was investigated through functional complementation experiments using the invertase-deficient yeast strain SEY2102. These results contribute to further understanding the roles of alkaline/neutral invertases in sucrose metabolism during the development of the pepper plant.

## 2. Results

### 2.1. Identification and Characterization of CaNINV Genes in Pepper

**Figure 1:**
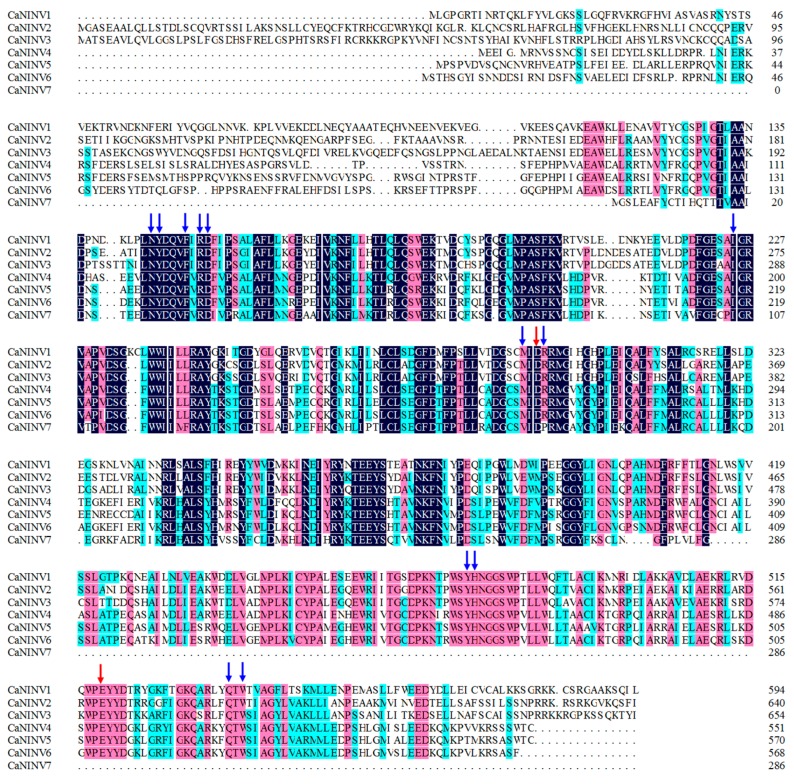
Alignment of the deduced amino acid sequences of the seven pepper neutral/alkaline invertases. Dark-blue shading, pinkish shading and light blue shading reflect 100%, 75% and 50% amino acid residues conservation, respectively. The catalytic residues and substrate-binding residues are depicted by red and blue arrows, respectively.

Based on the previously identified alkaline/neutral invertase sequences in *Arabidopsis thaliana*, BLAST analysis of the pepper genome database identified seven neutral/alkaline invertase genes in the pepper genome, which were named as CaNINV1 to 7. The full-length coding sequences of the *CaNINV* genes ranged from 861 bp (*CaNINV7*) to 1968 bp (*CaNINV3*). The size of deduced CaNINV proteins varied between 268 and 655 amino acids (aa), with an average of 552 aa. The molecular weight (Mw) varied from 32.49 to 73.57 kDa, and the theoretical pI of these genes ranged from 6.02 to 7.07 (Table 1). Computational analyses using ChloroP v.1.1, TargetP v.1.01, and Mitoprot II web tools showed that the resultant neutral/alkaline invertase proteins from three of the seven full-length cDNA clones might be located in cell organelles (CaNINV1 in mitochondria and/or plastid, CaNINV2, and 3 in plastid) (Table 1). Alignment analysis of amino acids showed that CaNINVs share 48.25–78.72% identity among all the family genes. CaNINVs have a higher similarity at the C-terminus, but a low similarity at the N-terminus of the amino acid sequence (Figure 1). CaNINVs contain multiple conserved residues that may play a key role in substrate-binding (using the numbering based on CaNINV1, the residues are D293 and E519) and catalytic function (using the numbering based on CaNINV1, the residues are N144, Y145, F148, R150, D151, I225, M291, R294, Y475, H476, Q537, and W539). 

### 2.2. Phylogenetic Analysis of CaNINV Genes

**Figure 2:**
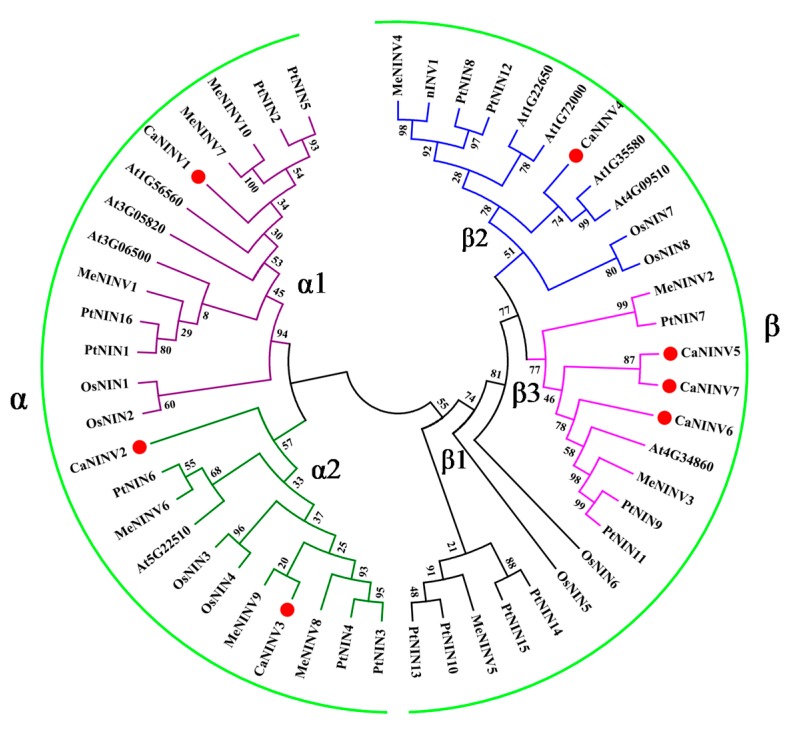
Phylogenetic analysis of alkaline/neutral invertase (NINV) proteins from *C. annuum*, *M. esculenta*, *P. trichocarpa*, *A. thaliana*, and *O. sativa*. The phylogenetic tree was constructed by Neighbor-Joining method (1000 bootstrap replicates) using Molecular Evolutionary Genetics Analysis 7.0 software. Red dots indicate the NINVs from *C. annuum*.

To determine the evolutionary relationship among the plant alkaline/neutral invertase (NINV) proteins, sequences of 51 NINV family members from *Capsicum annuum*, *M. esculenta*, *Populus trichocarpa*, *A. thaliana*, and *O. sativa* were analyzed using a Neighbor-Joining (NJ) phylogenetic tree. These plant NINV proteins were classified into two groups (α and β groups) (Figure 2). CaNINV1, 2, and 3 were classified in the α group. CaNINV4, 5, 6, and 7 were classified in the β group. In the α group, CaNINV1 had a close relationship with MeNINV7, 10, and PtNIN2, 5, and these genes were grouped into the α1 group. CaNINV2 and 3 shared 69.10% amino acid identity and showed a close relationship, so they were grouped into the α2 group, while CaNINV2 and MeNINV9 formed a clade. In the β group, CaNINV4 had a close relationship with At1G35580 and At4G09510, and these genes were grouped into the β2 group. CaNINV5, 6, and 7 had a close relationship and were grouped into the β3 group. In addition, CaNINV5 and 7 formed a clade, with 73.08% amino acid similarity. 

### 2.3. Structure Analysis and Chromosomal Distribution of the CaNINV Family Genes

**Figure 3:**
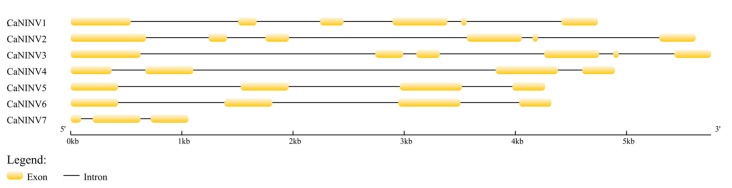
Exon–intron structure of the seven *NINVs* in pepper. Introns are shown as black lines, exons are shown as yellow boxes.

In order to gain further insight into the evolutionary relationships of *CaNINVs*, the exon–intron structure for each member of this family was analyzed. The number of exons in *CaNINV* genes ranged from three to six (Figure 3). Among the α group members, *CaNINV1*, *2*, and *3* had six exons, and their fifth exon was the smallest one among all of the exons in *CaNINVs*. Among the β group members, *CaNINV4*, *5*, and *6* had four exons, and the exon–intron structures of *CaNINV5* and *6* were similar. *CaNINV7* had only three exons.

**Figure 4:**
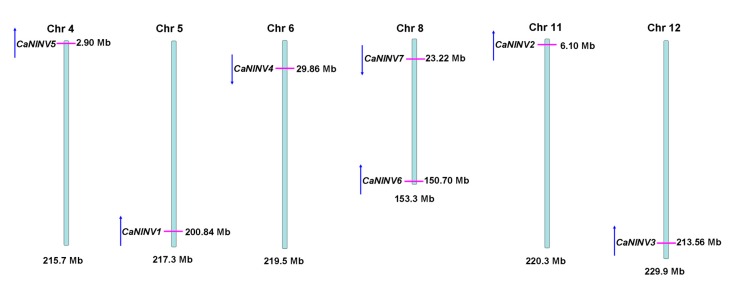
Chromosome localization of *CaNINV* genes from pepper. The positions of the *CaNINV* genes are shown as red lines. The genes orientation is shown as blue arrows.

The chromosomal distribution and orientation of *CaNINV* genes were also identified. The results showed that the seven *CaNINV* genes are mapped to six chromosomes of the pepper genome (Figure 4). No tandem duplication of the pepper *CaNINV* genes was found. *CaNINV5*, *1*, *2*, and *3* had the same orientation and are present on chromosome 4, 5, 11, and 12, respectively. *CaNINV4* was found to be present on chromosome 6, while *CaNINV6* and *7* were mapped to chromosome 8, with opposite orientation. 

### 2.4. Motif Distribution in CaNINV Proteins

**Figure 5:**
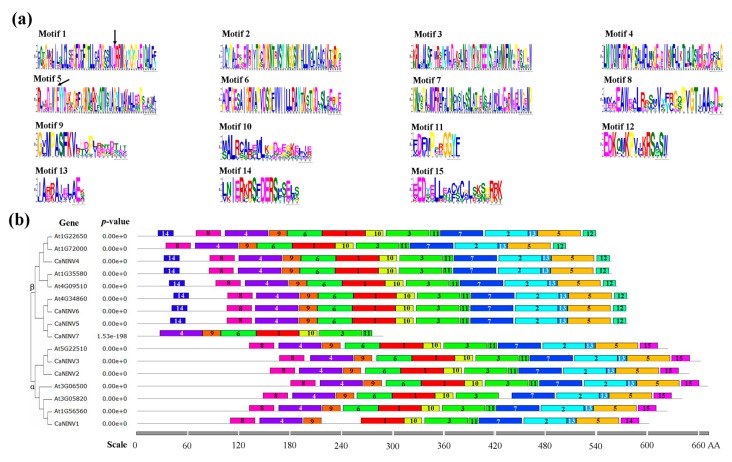
The conserved motifs of NINV proteins from *C. annuum* and *A. thaliana* according to the phylogenetic analysis. (**a**) The motif sequences in NINVs, which were identified by MEME. The catalytic residues are indicated by black arrow; (**b**) The motif distribution in NINVs. The NJ tree was constructed with full amino acid sequences of CaNINVs and AtNINVs using Muscle and MEGA7 software with 1000 bootstraps. Gray lines represent the non-conserved sequences, and each motif is indicated by a colored box and numbered at the box. The length of the motifs in each protein is shown proportionally.

To further investigate the structural features of CaNINV proteins in pepper, the putative motifs were analyzed according to their phylogenetic relationships, and 15 distinct motifs were identified. The distribution of these motifs was similar in CaNINVs and AtNINVs (Figure 5). Motifs 1, 3, 4, 9, 10, and 11 were widely distributed in all of the analyzed NINV proteins. Motifs 1 and 5 contain the catalytic residues. Most of the motifs classified in α or β CaNINV groups are similar, but the non-conserved sequences at N-terminus were longer in α group when compared with the β group. In addition, motif 15 was specifically distributed in α group, whereas motifs 12 and 14 were specifically distributed in β group. Especially in β group, several motifs of CaNINV7 were lost, in comparison with the other members of β group, at C-terminus (motifs 8 and 14) and N-terminus (motifs 2, 5, 7, 12, and 13). 

### 2.5. Three-Dimensional Structure of CaNINV Proteins

**Figure 6:**
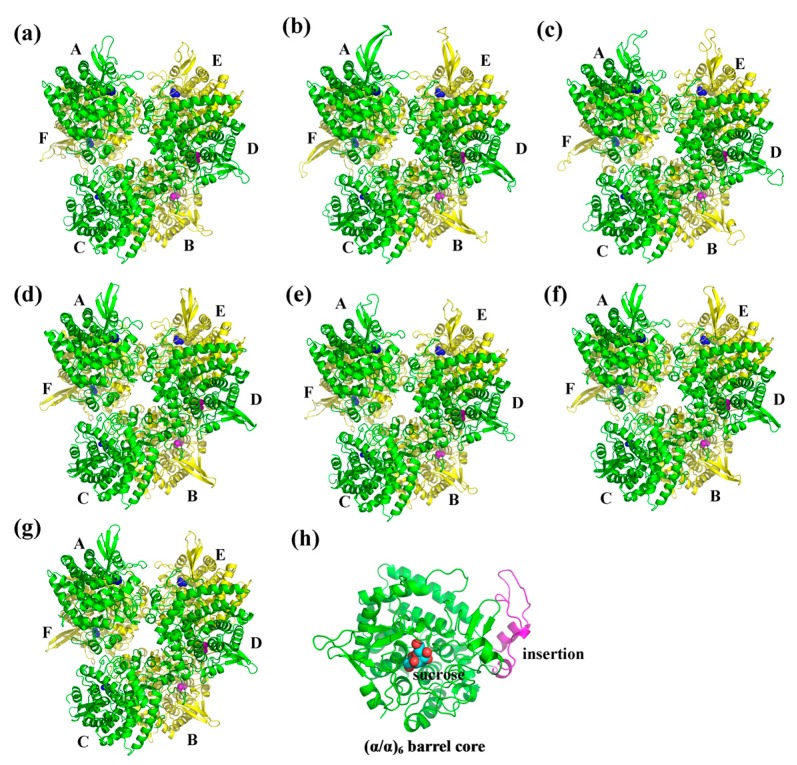
Cartoon representation of the predicted three-dimensional structure models of CaNINV1–6. (**a**) CaNINV1; (**b**) CaNINV2; (**c**) CaNINV3; (**d**) CaNINV4; (**e**) CaNINV5; (**f**) CaNINV6; and, (**g**) NINV protein from *Anabaena*; (**h**) the monomer of CaNINV1. The six subunits are sequentially labeled as A–F. The spherical structures indicate sucrose molecules. The image was generated using the PyMOL program (Schrödinger, Inc., New York, NY, USA).

**Figure 7:**
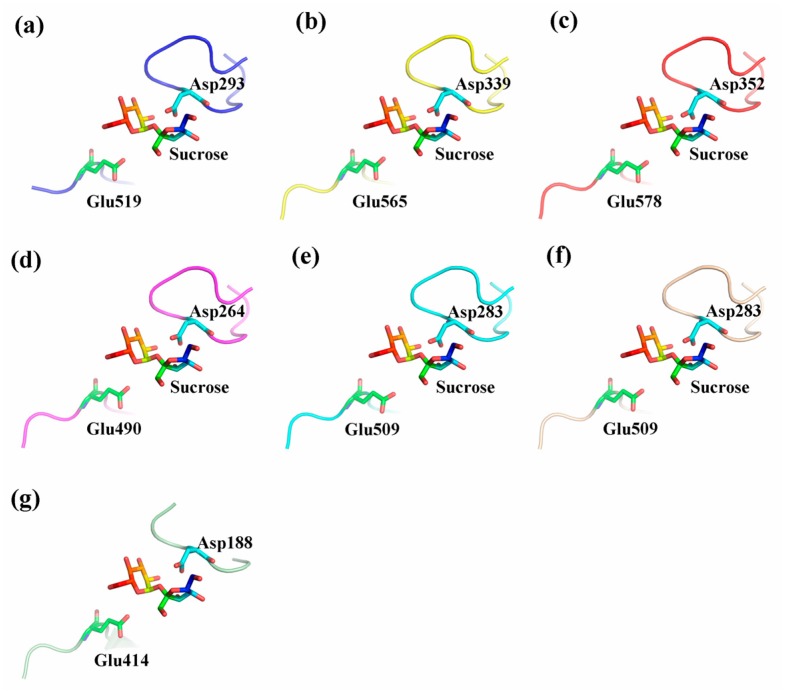
Predicted catalytic residues of CaNINV1–6 with sucrose molecules. (**a**) CaNINV1; (**b**) CaNINV2; (**c**) CaNINV3; (**d**) CaNINV4; (**e**) CaNINV5; (**f**) CaNINV6; (**g**) NINV protein from *Anabaena* (Protein Databank ID 5GOR). Colored stick structures indicate sucrose molecules. Green stick and blue stick structures represent the catalytic residues of Glu and Asp, respectively. The image was generated using the PyMOL program (Schrödinger, Inc., New York, NY, USA).

To obtain a reasonable theoretical structure of CaNINVs, protein homology modeling was performed using crystal structure of NINV protein from *Anabaena* (Protein Databank ID 5GOR) as a template, which shared 55.48%, 54.73%, 55.98%, 56.66%, 56.21%, and 54.33% sequence identity with CaNINV1–6, respectively. The 3D structure of CaNINV7 was not constructed by homology modeling. The predicted 3D models of CaNINV1–6 were validated using the QMEAN server (http://swissmodel.expasy.org/qmean/cgi/index.cgi) for model quality estimation. The total QMEAN scores (the estimated model reliability between 0 and 1) of the predicted 3D models for CaNINV1–6 were 0.65, 0.62, 0.60, 0.70, 0.68, and 0.68, respectively. These results indicated that all the sequences for CaNINV1–6 matched the homologous templates well on the server, implying that the models were reliable. CaNINV1–6 had very similar structural models. All CaNINVs can form a hexamer, wherein each monomer consists of a (α/α) 6-barrel core structure and an insertion of three helices. To predict the theoretical position of the sites for sucrose binding with CaNINVs, the primary models of CaNINVs were further structurally aligned with a model of the NINV protein from *Anabaena* (Protein Databank ID 5GOR) using the PyMOL program (Schrödinger, New York, NY, USA). The results showed that sucrose is predicted to bind in the catalytic pocket (Figure 6h) and the enzyme active sites (using the numbering based on CaNINV1, the residues were Asp293 and Glu519) of CaNINVs assumed the same orientation toward the sucrose molecules (Figure 7).

### 2.6. Expression Analysis of CaNINV Genes for Different Tissues and Developmental Stages

**Figure 8:**
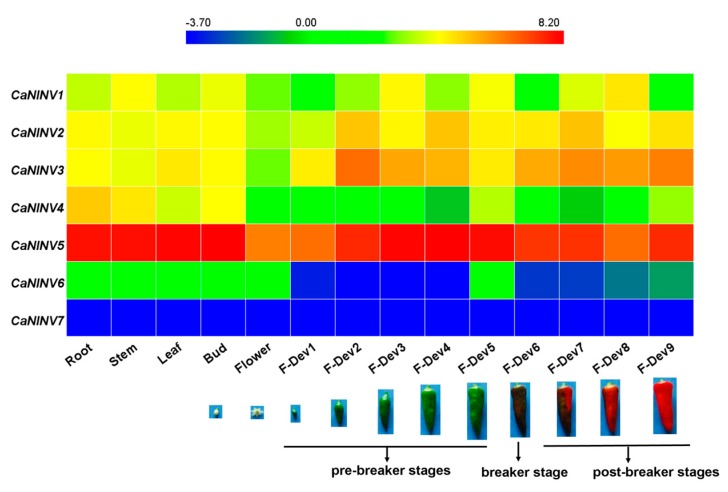
Expression profiles of seven pepper *CaNINV* genes for different tissues and developmental stages. The expression data were collect from the Illumina RNA-seq data obtained by pepper (cultivar Zunla-1) genome sequencing [30]. The images of the developmental stages of pepper fruit were cited from Qin et al. [30]. The FPKM values were log_2_ transformed and the heat map was generated using HemI (Heatmap Illustrator, version 1.0) software package. Bar at the top represents log_2_-transformed values. Genes highly or weakly expressed in the tissues are colored blue and red, respectively.

To establish the spatio-temporal expression patterns of *CaNINV* genes, the RNA-seq data from different tissues and developmental stages (root, stem, leaf, buds, flower, and nine stages of developing fruits) of pepper cultivar Zunla-1 [30] were used to generate a heat map. The expression pattern of each *CaNINV* gene was significantly different across different tissues and developmental stages (Figure 8). The expression of *CaNINV1* and *CaNINV6* were low in all of the tested tissues, and no expression of *CaNINV7* was detected. *CaNINV4* was mainly expressed in the root, and a lower expression was observed in fruits. The expression of *CaNINV2* and *CaNINV3* in fruits was higher than in rest of the tissues. Surprisingly, the expression of *CaNINV5* was the highest among all the *CaNINV* genes in all of the tested tissues. The expression of *CaNINV5* in the root, stem, leaves, and buds was similar, and was lowest in the flowers. During pepper fruit development, the expression of *CaNINV5* gradually increased at the pre-breaker stages, followed by a decrease at the breaker stages, and maintained a low level at the post- breaker stages.

### 2.7. Yeast Complementation of CaNINV5 

**Figure 9:**
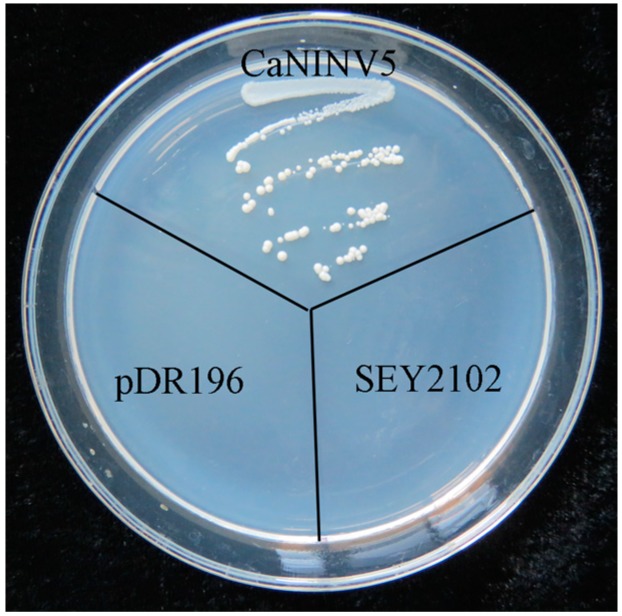
Complementation of the invertase-deficient strain SEY2102 with CaNINV5. *S. cerevisiae* strains were grown on SD media, which lacked uracil and had Suc as the sole carbon source, at 28 °C for 3 days. CaNINV5: the pDR196-*CaNINV5* vector was transformed into SEY2102; PRD196: the empty pDR196 vector was transformed into SEY2102; SEY2102: the mutant yeast cells without any vector transformation.

*CaNINV5* was the most highly expressed *CaNINV* gene across the different tissues and developmental stages of pepper. Therefore, its activity was further examined in the yeast triple mutant SEY2102, which lacks endogenous invertase activity. This yeast mutant is unable to grow on a medium with sucrose as the sole carbon source. First, the cDNA of *CaNINV5* was inserted into a yeast expression vector pDR196 to generate pDR196-*CaNINV5*. The results showed that yeast cells transformed with the empty pDR196 vector could not grow on the selection medium containing sucrose, whereas the yeast cells that were transformed with pDR196-*CaNINV5* could grow on this medium (Figure 9). This result suggested that CaNINV5 has an invertase activity.

### 2.8. Optimum pH Determination for CaNINV5

**Figure 10:**
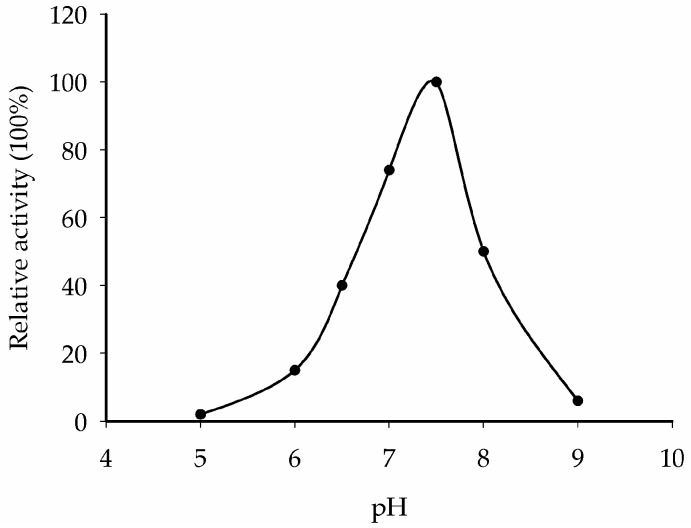
Representation of the pH dependence of alkaline/neutral invertase activity of CaNINV5.

To confirm if CaNINV5 proteins indeed encode alkaline/neutral invertase, we extracted the crude proteins expressed by *CaNINV5* gene from *S. cerevisiae* strain SEY2102. The enzyme activities of CaNINV5 at different pH values (pH 5.0–9.0) were assayed. The results show that the optimum pH of CaNINV5 is ~7.5 (Figure 10), indicating that CaNINV5 is an alkaline/neutral invertase.

## 3. Discussion

### 3.1. Identification and Characterization of CaNINV Genes

Alkaline/neutral invertases play an important role in sugar metabolism during fruit development [31]. In addition, alkaline/neutral invertases have been reported to be involved in carbohydrate metabolism during the development of pepper fruits [32,33]. However, no further information is available about the alkaline/neutral invertase gene family in pepper. Previous studies have reported that the members of the alkaline/neutral invertase gene family vary among plant species. For instance, it has sixteen members in *Populus trichocarpa* [16], eleven members in *Manihot esculenta* Crantz, eight members in *Oryza sativa* [15], and nine members in *A. thaliana* [18]. In the present study, seven alkaline/neutral invertase genes (*CaNINV1–7*) were found in pepper (Table 1). *P. trichocarpa* have more NINVs than *M. esculenta*, *O. sativa*, *A. thaliana*, and *C. annuum* due to the salicoid genome duplication event [16]. CaNINVs (32.49–73.57 kDa) have larger molecular weight range than MeNINVs (48.6–79.7 kDa) from *M. esculenta* [17]. All of the CaNINV proteins were found to contain multiple conserved residues (Figure 1), which are consistent with the reported NINVs in other plants, such as At1G56560 from *A. thaliana* [34]. While, CaNINV7 is lost a catalytic residues and two substrate-binding residues at C-terminus. Phylogenetic analysis of the 51 NINV proteins from five plants showed that the CaNINVs could be classified into α and β groups, among which, the α group is predicted to be located in the organelles (Table 1, Figure 2). This result is consistent with the evolutionary characteristics of invertases [8]. The α group members have similar exon–intron structures and motif distributions. In contrast, CaNINV7 appears to have lost amino acid sequences at both C-terminus and N-terminus, and has different exon–intron structures and motif distribution compared with the other members in β group (Figure 1, Figure 2 and Figure 4). when compared to the motif distributions of NINVs from *A. thaliana* and *C. annuum* may reveal their natural variability. The α2 group members (CaNINV2, CaNINV3, and At5G22510) all have 15 distinct motifs, while the α1 group members CaNINV1 and At3G05820 are lost motif 6 and motif 11, respectively. The β group members CaNINV7 and At1G7200 are both lost motif 14, while CaNINV7 lost more motifs (Figure 2 and Figure 4). Similarly, it was found that MeNINV5 and MeNINV9 from *M. esculenta* are lost several motifs [17]. Recently, the first crystal structure of alkaline/neutral invertase (InvA) from *Anabaena* was reported, which was proposed to be the ancestor of the modern plant alkaline/neutral invertase [34]. In this study, the 3D structural models of CaNINV1–6 showed that all of these proteins can form a hexamer, wherein each monomer consists of an (α/α) 6-barrel core structure and an insertion of three helices, and sucrose is predicted to bind in the catalytic pocket (Figure 6). The enzyme active sites of CaNINVs assumed the same orientation toward the sucrose molecules (Figure 7). These structures are typical of InvA proteins [34]. The results of the sequence and 3D structure analyses of CaNINV1–6 suggest that all these alkaline/neutral invertase members from pepper can catalyze the irreversible hydrolysis of sucrose to glucose and fructose. 

### 3.2. Differential Expression and Enzymatic Activities of CaNINVs

The tissue-specific expression patterns of *CaNINVs* could provide a basis for understanding their functions in pepper plant development. The expression patterns of *CaNINV1–7* in various organs and tissues were examined. *CaNINV1–5* were widely expressed in most plant tissues. *CaNINV2* and *CaNINV3* were highly expressed in the fruits and showed a lower expression in other tissues, while the proteins of these two genes were both predicted to localize in the plastid. The plastid in pericarp tissue is important for pepper fruit development, and chloroplasts can be transformed to chromoplasts during ripening [35]. This result suggests that these two genes might play a key role in plastid development. The expression of *CaNINV1*, *CaNINV6*, and *CaNINV7* was low or undetectable. The expression of some plant *NINV* genes has been reported to be affected by abiotic stress. In *A. thaliana*, *NINV* gene *AtCYT-INV1* is involved in osmotic stress-induced inhibition on lateral root growth [36]; the expression of *A/N-InvA* (a mitochondrial NINV) and *A/N-InvG* (a cytosolic NINV) was induced under H_2_O_2_ treatment, and was involved in oxidative stress defense connection [37]. The expression of *NINV* gene *Ta-A-Inv* is induced in response to osmotic and cold stress in mature primary wheat leaves [38]. Therefore, we speculate that the expression of *CaNINV1*, *CaNINV6*, and *CaNINV7* might be related to some stress conditions. *CaNINV4* was mainly expressed in the root. Rice NINV gene *SRT5* has been reported to have a role in carbon and energy supply during early root development [24]. Therefore, we speculate that *CaNINV4* might play a role in sucrose metabolism during pepper root development. Interestingly, *CaNINV5* was the most active gene among the *CaNINVs* in all of the tested tissues. During pepper fruit development, the expression of *CaNINV5* gradually increased at the pre-breaker stages, then decreased at the breaker stages, and maintained a low level at the post-breaker stages. This expression pattern of *CaNINV5* is consistent with the previously reported trend of alkaline/neutral invertase activity [27]. Further investigation showed that CaNINV5 could complement an invertase-deficient yeast strain to grow on a medium with sucrose as the sole carbon source, indicating that CaNINV5 can catalyze the hydrolysis of sucrose. The optimum pH for the enzyme activity of alkaline/neutral invertases is typically between pH 6.5 and 8.2 [5,14]. To confirm if CaNINV5 indeed encodes an alkaline/neutral invertase, the enzyme activity of CaNINV5 was assayed at different pH values. The results showed that the optimum pH of CaNINV5 is ~7.5, indicating that CaNINV5 is an alkaline/neutral invertase. This result indicates that *CaNINV5* plays a key role in the sucrose catabolism and the development of pepper fruit.

## 4. Materials and Methods

### 4.1. Plant Materials

The leaves were collected from pepper cultivar “Zunla-1” (*Capsicum annuum* L.) plants (provided by the Pepper Institute, the Zunyi Academy of Agricultural Sciences, Zunyi, China), which were planted in the field and were used for gene cloning. Fresh leaves from the plants were collected and frozen in liquid nitrogen for subsequent RNA isolation.

### 4.2. Identification and Sequence Analysis of CaNINV Proteins in Pepper

To identify pepper genes encoding CaNINV proteins, the alkaline/neutral invertase proteins AtINV1–9 in *Arabidopsis thaliana* were used to do BLASTP search in pepper genome databases (http://peppersequence.genomics.cn/page/species/index.jsp, release 2.0) with an expected value (e-value) cut-off of 0.01 [8]. All of the protein sequences obtained were confirmed by the Pfam (http://pfam.xfam.org/search) and SMART (http://smart.embl-heidelberg.de/). The deduced amino acids of CaNINVs were analyzed by multiple alignment using DNAman 6.0 software (Lynnon Biosoft, Quebec City, QC, Canada). Molecular weight (MV) and theoretical isoelectric point (PI) of CaNINV proteins were computed by ProtParam (http://web.expasy.org/protparam/). The subcellular location of CaNINV proteins were predicted using ChloroP (http://www.cbs.dtu.dk/services/ChloroP/, version 1.1), TargetP (http://www.cbs.dtu.dk/services/TargetP/, version 1.1) and Mitoprot II (https://ihg.gsf.de/ihg/mitoprot.html, version 1.101) web tools. The catalytic residues and substrate-binding residues of CaNINV proteins are depicted correspond with the residues of *Anabaena* alkaline invertase InvA proposed by Xie et al. [34].

### 4.3. Phylogenetic Analyses

A total of 51 NINVs from *C. annuum*, *M. esculenta*, *P. trichocarpa*, *A. thaliana*, and *O. sativa* were aligned using the MUSCLE program. The resulting alignment was manually optimized by removing unaligned residues, and the phylogenetic tree was constructed and drawn using Molecular Evolutionary Genetics Analysis Version 7.0 (MEGA7, Tokyo Metropolitan University, Tokyo, Japan) by the Neighbor-Joining (NJ) method. The branching reliability was assessed by the bootstrap re-sampling method using 1000 bootstrap replicates.

### 4.4. Exon–Intron Structure Analysis and Chromosomal Mapping 

In order to display the structure of introns and exons of *CaNINV* genes, the cDNA sequences of *CaNINV* genes were aligned with the corresponding genomic DNA sequences from the pepper genome database (http://peppersequence.genomics.cn/page/species/index.jsp, release 2.0). The Gene Structure Display Server (GSDS) program (http://gsds.cbi.pku.edu.cn/, version 2.0) was used to visualize the gene structure [39]. The genomic position of the *CaNINV* genes and the total length of each chromosome were obtained from the pepper genome database. Subsequently, the *CaNINV* genes were manually mapped onto chromosomes.

### 4.5. Conserved Motif Analysis

The program MEME was used to predict the potential motifs in the protein sequences of alkaline/neutral invertases in *C. annuum* and *A. thaliana*. MEME was run online (http://meme-suite.org/tools/meme, version 4.12.0) using the following parameters: distribution of motif occurrences (zero or one per sequence), number of different motifs (15), minimum motif width (6), and maximum motif width (50).

### 4.6. Prediction of Three-Dimensional Structure of the CaNINV Proteins

Full-length amino acid sequences of the seven pepper CaNINV proteins were submitted to the Swiss-Model server (http://beta.swissmodel.expasy.org/) to predict their three-dimensional structure, and the crystal structure of NINV protein from *Anabaena* (Protein Databank ID 5GOR) was used as a template. The three-dimensional structure and catalytic residues of CaNINVs were displayed using the Pymol software (Delino Scientific, San Carlos, CA, USA).

### 4.7. Pepper RNA-Seq Data Analysis

Spatio-temporal (root, stem, leaves, buds, flowers, and nine developmental stages of fruit) expression profiling of pepper *CaNINV* genes was done using the Illumina RNA-seq data from pepper (cultivar Zunla-1) genome sequencing [30]. The expression values of pepper *CaNINV* genes were calculated as fragments per kilobase of transcript per million fragments mapped (FPKM). The HemI (Heatmap Illustrator, version 1.0, Huazhong University, Wuhan, China) software packages were used to construct a heat map to visualize the expression profiling based on log_2_-transformed RPKM values. 

### 4.8. Cloning of Full-Length CaNINV5

Full-length cDNAs of the *CaNINV5* gene were isolated by reverse transcription PCR using gene-specific primers: CaNINV5-F (5′ ATGCCTAGCCCTGTGGATGTGTC 3′) and CaNINV5-R (5′ TTAACAGGTCCAAGAAGCAGATCTT 3′). Total RNA was isolated from pepper leaves using RNAplant Plus reagent (TianGen, Beijing, China). First-strand cDNA was synthesized from 3 μg of total RNA sample using Anchored Oligo(dT)_18_ Primer primers and the TransScript First-Strand cDNA Synthesis SuperMix Kit (TransGen, Beijing, China), following the manufacturer’s instructions. The PCR fragment was cloned into the pEASY^®^-T3 cloning vector (TransGen, Beijing, China) and the sequencing of independent clones was performed on both of the strands by Sangon Biological Engineering Technology and Services (Shanghai, China).

### 4.9. Yeast Complementation and Enzymatic Analysis of CaNINV5

For the *S. cerevisiae* complementation assays, an invertase-deficient strain SEY2102 was kindly provided by Liu, et al. [23]. The yeast shuttle vector pDR196, containing *URA3* as a selective marker, was used for transformation. The cDNA of *CaNINV5* was inserted into the EcoRI/XhoI sites within pDR196, and the new plasmid was verified by sequencing and designated as pDR196-*CaNINV5*. pDR196 and pDR196-*CaNINV5* vectors were transformed into the SEY2102 strain using PEG/LiAc method. Transformants were selected on synthetic dropout (SD) medium without uracil. The function of CaNINV5 was confirmed by the growth status of the transformant strain on SD medium, with sucrose as the sole carbon source.

SEY2102 yeast cells, transformed with pDR196–*CaNINV5*, were grown in 30 mL of −URA/sucrose liquid media for 3 days. Yeast cells were spun down and yeast protein was extracted by vortexing with glass beads. Protein contents were measured by Bradford’s method. Crude enzyme extracts were used for determination of enzyme activities of CaNINV5 at different pH values. Enzyme activity analysis was carried out as in Liu, et al. [23]. 

## Figures and Tables

**Table 1:** Basic information of seven pepper alkaline/neutral invertase genes (*CaNINVs*).

Gene Name	Gene ID	ORF Length (bp)	Protein Length (aa)	MW (kDa)	PI	Localization
*CaNINV1*	Capana05g002151	1788	595	67.84	6.04	Mitochondria and/or plastid
*CaNINV2*	Capana11g000243	1926	641	72.77	6.94	Plastid
*CaNINV3*	Capana12g002379	1968	655	73.57	6.18	Plastid
*CaNINV4*	Capana06g001364	1656	551	62.98	6.07	
*CaNINV5*	Capana04g000213	1713	570	65.18	6.02	
*CaNINV6*	Capana08g002679	1707	568	65.09	6.35	
*CaNINV7*	Capana08g000259	861	286	32.49	7.07	
